# Genome survey and chromosome number determination of *Polygala fallax* (Polygalaceae), an endemic medicinal plant from southern China

**DOI:** 10.3897/compcytogen.20.184176

**Published:** 2026-04-15

**Authors:** Lin Fu, Ziyue Liu, Lina Han, Ting Zhang, Yan Jiang, Denis A. Krivenko, Jin Hu, Hongfeng Chen, Lei Duan

**Affiliations:** 1 State Key Laboratory of Plant Diversity and Specialty Crops/Guangdong Provincial Key Laboratory of Applied Botany, South China Botanical Garden, Chinese Academy of Sciences, Guangzhou 510650, China Siberian Institute of Plant Physiology and Biochemistry, Siberian Branch, Russian Academy of Sciences Irkutsk Russia; 2 South China National Botanical Garden, Guangzhou, 510650, China South China Botanical Garden, Chinese Academy of Sciences Guangzhou China; 3 Guangdong Liangtian Agriculture & Forestry Technology Co., Ltd., Qingyuan, 511899, China University of Chinese Academy of Sciences Beijing China; 4 University of Chinese Academy of Sciences, Beijing, 100049, China South China National Botanical Garden Guangzhou China; 5 Siberian Institute of Plant Physiology and Biochemistry, Siberian Branch, Russian Academy of Sciences, Irkutsk 664033, Russia Guangdong Liangtian Agriculture & Forestry Technology Co., Ltd. Qingyuan China; 6 Guangdong Eco-Engineering Polytechnic, Guangzhou, 510520, China Guangdong Eco-Engineering Polytechnic Guangzhou China

**Keywords:** Chromosome number, genome size, genome survey, *Polygala fallax*

## Abstract

*
Polygala fallax* Hemsley, 1886 is a perennial herb of the family Polygalaceae. Because of its antioxidant, anti-aging, anti-inflammatory, and antibacterial properties, it is regarded as a traditional medicinal plant in China with high economic value. Although this species has significant medicinal and health benefits, cytological research on it remains very limited. In the present study, the genome size and chromosome number of *P. fallax* were determined through k-mer analysis, flow cytometry, and cytogenetics. Firstly, a genome survey was conducted using Illumina NovaSeq 6000 DNA sequencing. K-mer analysis revealed that the genome size of *P. fallax* was approximately 2199.64 Mb (ca. 2.20 Gb), the heterozygosity rate was about 1.11%, and the proportion of repeated sequences was about 73.61%. As for flow cytometry analysis, *Solanum lycopersicum* Linnaeus, 1753 was used as internal standard species, and the 2C-DNA value of *P. fallax* is 2.49 Gb. We counted the chromosome number of *P. fallax* using a common cytogenetic method, and the result showed a chromosome number of 2n = 40. This comprehensive study offers a foundation for subsequent whole-genome sequencing of *P. fallax*, contributing valuable insights into its genetic characteristics and providing genomic information for further research on the synthesis mechanisms of its medicinal secondary metabolites, species conservation, and sustainable utilization.

## Introduction

**Figure 1.**
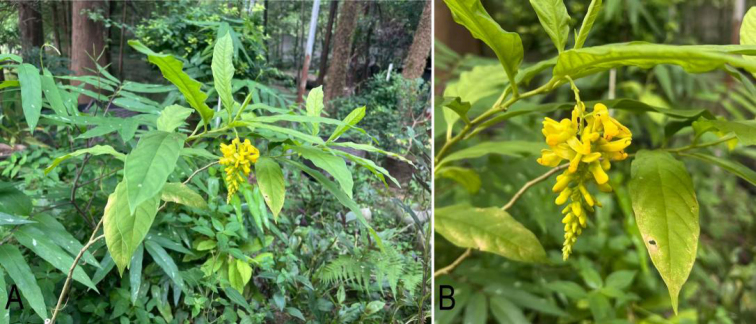
The plant and flowers of *Polygala fallax*. **A** plant in the primary habitat **B** raceme with yellow flowers.

*Polygala fallax* Hemsley, 1886, a member of the family Polygalaceae, is an endemic species in southern China, mainly distributed in Fujian, Guangdong, Guangxi, Guizhou, Hunan, Jiangxi and Yunnan Provinces. It is a shrub or small tree with stout roots, gray-green branchlets, lanceolate to elliptic-lanceolate leaves and yellow flowers in racemes (Fig. 1), and growing in forests, streamsides and moist habitats in valleys at altitudes of 400–1700 m (Chen et al. 2008). It is a perennial plant that has been widely used in traditional Chinese medicine for treating lumbar and leg pain resulted from strain, as well as acute and chronic hepatitis. Its main active components, including flavonoids, polysaccharides, volatile oils, and saponins, exhibit pharmacological activities such as antioxidant, anti-aging, anti-inflammatory, and antibacterial effects (Xu et al. 2006; Yao et al. 2020). Recent research found it has significant pharmacological effects in enhancing stress resistance, lowering lipid level, exhibiting anti-hepatocellular carcinoma activity, suppressing inflammation, improving myocardial ischemia, and facilitating recovery from the liver tissue (Klein et al. 2012; Wang et al. 2022). Ning and Dela Cruz (2023) reported that *P. fallax* might delay aging in rat through down-regulating the phosphorylation of *PI3K*, *AKT*, *mTOR* and *S6K1*, key proteins involved in PI3K/AKT/mTOR signaling pathway and has a promising application in anti-aging effect.

Due to the wide utilization as traditional medicine in southern China, natural resources of *P. fallax* have been extensively exploited, making its wild individuals over-collected. Genomic study is an important strategy to provide comprehensive data on individuals’ genetic variation, offering the most complete information for researches on plant growth, development, evolution, and adaptation. However, most of current studies on *P. fallax* focus on its pharmacological components and activities, genomic study is still limited until now.

Before conducting whole-genome sequencing, it is necessary to understand the characteristics of the genome, such as heterozygosity, repetitive sequences, and GC content, in order to determine an efficient and accurate assembly strategy for subsequent research. Genome survey can provide preliminary information on genome structure, such as genome size, heterozygosity, and repeat contents. Genome size estimated by flow cytometry and chromosome number counting are also necessary analyses for studying genomic characterization. These methods can evaluate genome size from different perspectives, ensuring the sequencing progress and providing reliable support for subsequent genome assembly.

*Polygala* Linnaeus, 1753 is the largest genus within the family Polygalaceae, including about 600 taxa around the world (Chen et al. 2008). Genome size is one of the most extensively studied topics in plant systematics and angiosperm evolution (Jang et al. 2018), while within the family, only the genome of *P. tenuifolia* Willdenow, 1802 has been reported till now, of which the assembled genome is 769.62 Mb and the estimated genome size is 798.58 Mb (Meng et al. 2023). Only 11 species’ genome sizes in *Polygala* have been recorded in the “Plant DNA C-value database” (Leitch et al. 2019; https://cvalues.science.kew.org/; release 7.1, April 2019), ranging from 0.84 pg (0.82 Gb, 2C-DNA value) in *P. amara* subsp. *brachyptera* (Chodat, 1889) Hayek, 1906 to 3.10 pg (3.03 Gb, 2C-DNA value) in *P. paucifolia* Willdenow, 1802, generally larger than *P. tenuifolia*.

According to the combined data in the “Chromosome Counts Database”, CCDB: https://ccdb.tau.ac.il/ (Rice and Mayrose 2023) and new cytological studies (e.g. Shi and Niu 2017), nearly 100 *Polygala* species’ chromosome numbers have been reported, with different ploidy status and primary basic chromosome number. Variations in chromosome numbers are considered as a major source of evolution and diversification in plants (Guerra 2008; Weiss-Schneeweiss and Schneeweiss 2013; Choi et al. 2019; Kang et al. 2021; Kim et al. 2024). Lewis and Davis (1962) found four primary basic chromosome numbers in *Polygala*: x = 6(12), 7(14), 8 and 10 and x = 17, 19 and 23 as secondary base. A perusal of the chromosomal data for the various species of Polygalaceae reveals a greater diversity in the chromosome numbers with 2n = 14, 16, 24, 28, 30, 32, 34, 36, 38, 40, 42, 46, 52, 56, 68, 72 and 96 (Bolkhovskikh et al. 1969; Moore 1973).

At present, comprehensive cytogenetic data, including chromosome number and genome size of *P. fallax* has not been reported. Therefore, the objectives of this study were to estimate the genome size estimation and chromosome numbers of *P. fallax* through flow cytometry, k-mer analysis, and chromosome counts. The study revealed some basic genomic features of the genome, offering a foundation for subsequent whole-genome sequencing of *P. fallax*, and providing genomic information for further research on the synthesis mechanisms of its medicinal components, species conservation, and sustainable utilization.

## Material and methods

### Plant materials

The plant material of *Polygala fallax* were collected from natural populations in Ruyuan County, Guangdong Province, China (24.91255589°N, 113.03523594°E, alt. 890 m), then cultivated in the South China Botanical Garden, Chinese Academy of Sciences. In total, five individual plants were used for chromosome study. The voucher specimens were deposited in the Herbarium of South China Botanical Garden (IBSC).

### Methods

#### Genome size estimation

Flow cytometry analysis is a characterization method in cytology, which determines the amount of nuclear DNA content in cells by measuring the fluorescence intensity proportional to DNA content, thereby offering a macroscopic viewpoint for observing genomic material (Mgwatyu et al. 2020). Three fresh plants of *P. fallax* were collected for flow cytometry analysis, and we used *Solanum lycopersicum* Linnaeus, 1753 (2C-DNA value = 0.90 Gb) as the internal reference standard (The Tomato Genome Consortium 2012). Seeds of tomato were provided by the Kunming institute of botany, Chinese Academy of Sciences, and one plant individual was used as material sources in the analysis.

Fresh leaf material of each studied individual was chopped with a razor blade, mixing with leaf material of an internal standard in LB01 buffer, and standing for 10 min. The resulting liquid with chopped plant material was filtered and the nuclei suspension was stained with propidium iodide (50 µg/mL, Sigma-Aldrich) and 50 µg/mL of ribonuclease A (RNase A, Boehringer), kept on ice for 0.5–1 h in dark environment (Doležel et al. 2007). The concentrations of the sample suspension is 2 × 10^5^ particles/mL. Measurements were carried out with a BD FACSCalibur (BD Biosciences, San Jose, CA, USA). The fluorescence intensity of propidium iodide was detected by a standard 488 nm blue light excitation. One run can be done per preparation, in which 10000 particles were measured (CV% was controlled within 5%). The generated data was analyzed using Modifit 3.0 software (Becton Dickinson). The peak values corresponding to the G0/G1 phase of both the determination samples and internal standard were obtained and recorded. The DNA content of our samples was calculated according to the following formula: Sample DNA content = Reference DNA content × (sample 2C mean peak position/reference 2C mean peak position). Three independent replicates (runs) of each individual were analyzed.

#### Genome survey

Fresh leaves (over 50 g) were collected, cleaned with 75% ethanol, then quick-frozen with liquid nitrogen for genome survey. The analysis was conducted on Illumina NovaSeq 6000 platform. The genomic DNA was extracted according to the CTAB protocol (Doyle and Doyle 1987), and the purity and concentration of DNAs were tested. A paired-end library was constructed and sequenced using the Illumina NovaSeq 6000 platform to generate approximately 50× coverage of the estimated genome. Raw reads were trimmed using FastP v0.21.0 with the key parameters “–detect_adapter_for_pe” (Chen et al. 2018), and quality control of raw sequence data was launched using FastQC v0.11.9 with the key parameters “-o .” (Lo et al. 2014). The reads were filtered before assembly to ensure that a pair of paired-end reads had more than 90% of bases with quality ≥ Q30. Genome size, heterozygosity, and repeat content were estimated by analyzing the 19-mer frequency distribution of the clean reads with Jellyfish and GenomeScope (Marçais and Kingsford 2011; Vurture et al. 2017). K-mer depth distribution was counted and the peak value of the depth distribution was identified. The mean k-mer depth equals the peak value of the k-mer depth distribution as the depth of the k-mer coverage following a Poisson distribution. The k-mer count distribution from Jellyfish was used to produce a report and several informative plots describing the genome properties by GenomeScope 2.0 (Vurture et al. 2017). The heterozygous ratio and repeat sequence content were obtained with GCE software (Liu et al. 2013).

#### Chromosome number

Young root tips (1–2 cm long) from five individual plants were collected between 10:00–11:00 a.m. Root tip meristems were pretreated with 0.01% aqueous colchicine solution for 6 h at room temperature, fixed in 3 : 1 (v/v) absolute ethanol/glacial acetic acid for 24 h, then transferred to 70% ethanol and stored at –4 °C. For chromosome counting, fixed root tips were hydrolysed in 1M HCL at 60 °C for 50 min in a water bath, rinsed three times in distilled water and stained with carbol fuchsin for 5 min. Meristems were subsequently excised and squashed for microscope observations. The mitotic metaphase chromosomes were observed and recorded in an Olympus BX-43 microscope at 100× magnification with an Olympus DP26 camera, and the best cell images were selected for processing with Adobe Photoshop.

### Data availability

The newly generated Illumina NovaSeq 6000 paired-end short-read sequencing data used for the *Polygala fallax* genome survey (i.e., the Illumina short-read dataset underpinning the k-mer–based genome profiling described in this study) have been deposited in CNGB database and are publicly available at https://ngdc.cncb.ac.cn/gsa/browse/CRA039476.

## Results

### Genome size estimation

**Figure 2.**
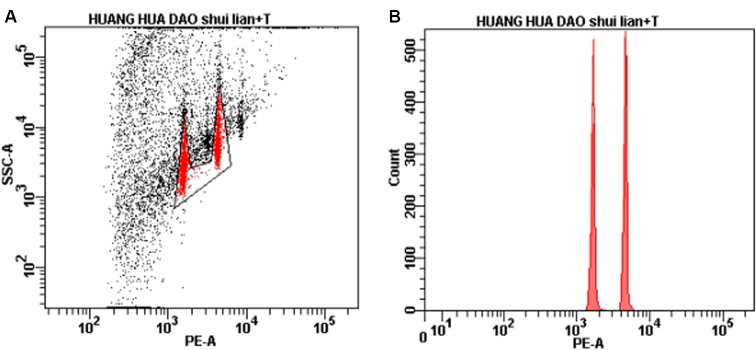
Flow cytometry analysis results of *Polygala fallax* and *Solanum lycopersicum***A** Scatter plots of mixed samples of *P. fallax* and *S. lycopersicum* (after PI staining). **B** Histogram of mixed samples of *P. fallax* and *S. lycopersicum*.

For flow cytometry analysis, *Solanum lycopersicum* (2C-DNA value = 0.90 Gb) was used as the internal reference standard (The Tomato Genome Consortium 2012). Flow cytometry results of *Polygala fallax* is shown in Table 1 and Fig. 2. The fluorescence intensity of *S. lycopersicum* was 1563 (left peak of the histogram in Fig. 2) with the CV 3.9%, and that of *P. fallax* was 4323 (right peak of the histogram in Fig. 2) with the CV 3.6%. The fluorescence intensity ratio of *P. fallax* to the *S. lycopersicum* is 2.77, which suggests that the nuclear DNA content (2C-DNA value) of *P. fallax* is approximately 2.49 Gb.

**Table 1.** Genome size of *Polygala fallax* and *Solanum lycopersicum* determined by flow cytometry.

**Species**	**Events**	**Fluorescence intensity**	**CV (%)**	**Genome size (Gb)**	**Mean value** ± **SD of genome size (Gb)**
* Polygala fallax*	1465	4323	3.6	2.49	2.49 ± 0.97
* Solanum lycopersicum*	1311	1563	3.9	0.90	

### Genome survey

Using paired-end sequencing of the genome with the Illumina NovaSeq 6000 platform, 110.40 Gb raw data were obtained, then we applied FastP filtering to remove the adapters and 109.79 Gb clean data was generated. The Q20 ratio of the sequencing data was above 99%, and the Q30 ratio was over 97% (Table 2), indicating a high quality of the sequencing data. The genome’s GC content was 38.3%, which is moderate and it is unlikely to cause GC bias.

**Table 2.** Statistics of genome sequencing data of *Polygala fallax*.

**Type**	**Number of read**	**Number of base**	**GC content (%)**	**Q20 (%)**	**Q30 (%)**
raw data	736,021,030	110,403,154,500	38.36	99.44	97.30
clean data	732,956,988	109,792,276,089	38.30	99.58	97.57

In Totall 50,000 sequences were selected randomly from the clean data and compared for homology in the NT library using BLASTn (https://ftp.ncbi.nlm.nih.gov/blast/executables/blast+/2.11.0/). The top 8 species belong to the family Polygalaceae and Fabaceae, and *P. fallax* has the highest sequence alignment rate of 0.27% (Table 3). Due to the unknown genomic information of *P. fallax* and the scarcity of gene annotations in the NT database, the comparison percentage is very low. According to the results, the sample library was aligned to DNA of closely related species, indicating that there was no significant exogenous contamination in the library, and the library construction and sequencing were successful.

**Table 3.** Genome sequence data NT library comparison of *Polygala fallax*.

**Species**	**Family**	**Kingdom**	**Reads**	**Percentage (%)**
* Polygala fallax*	Polygalaceae	Viridiplantae	135	0.27
* Glycine soja*	Fabaceae	Viridiplantae	128	0.26
* Cicer arietinum*	Fabaceae	Viridiplantae	110	0.22
* Trifolium occidentale*	Fabaceae	Viridiplantae	92	0.18
* Trifolium striatum*	Fabaceae	Viridiplantae	90	0.18
* Trifolium repens*	Fabaceae	Viridiplantae	90	0.18
* Lotus japonicus*	Fabaceae	Viridiplantae	79	0.16
* Polygala arillata*	Polygalaceae	Viridiplantae	72	0.14

**Figure 3.**
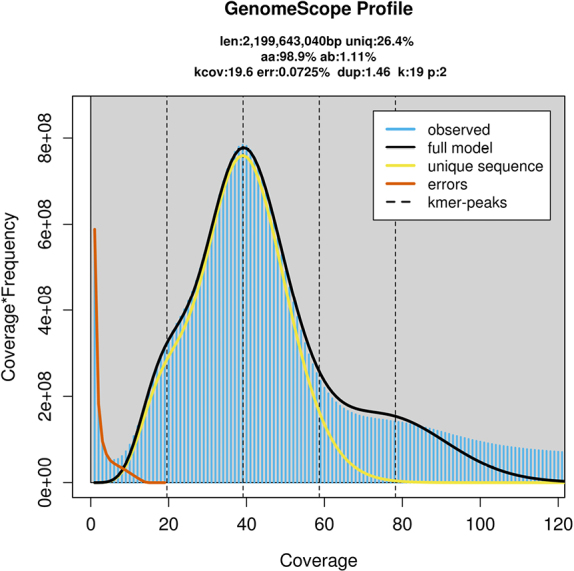
The 19 k-mer distribution of *Polygala fallax*.

Using 109.79 Gb of effective sequencing data, K = 19 was selected for analysis. After processing and analyzing the sequencing reads using Jellyfish, the total number of k-mers was 85,526,950,325. The k-mer frequency distribution was plotted using GenomeScope v.2, and the k-mer frequency distribution curve is shown in Fig. 3, with the horizontal axis representing k-mer depth and the vertical axis representing k-mer frequency. The figure shows that the k-mer distribution curve has a main peak around the depth of 39.2, a heterozygosity peak near 19–20 (approximately half coverage of the main peak) and a duplication peak near 80 (approximately two times coverage of the main peak), consistent with a diploid genome. The tailing phenomenon of the duplication peak indicates the presence of repetitive sequences and a relatively big genome. Although the heterozygosity peak is not prominent as the duplication peak, GenomeScope v.2 further estimated the genome heterozygosity by quantifying the proportion of the “heterozygous peak”, and repetitive sequence content of *P. fallax*, finding a genome heterozygosity of 1.11% (> 1%) and a repetitive sequence content of 73.61%, a high repeat sequence ratio (> 50%).

Based on the total number of k-mers and the main peak k-mer depth of 39.2, the preliminary calculated genome size of *P. fallax* was 2199.64 Mb (ca. 2.20 Gb), and the sequencing depth was estimated to be 49.91×. Therefore, the genome size obtained through k-mer analysis is slightly smaller but close to the result estimated by flow cytometry (2.49 Gb), suggesting a relatively high reliability of the measurement results.

### Chromosome number

**Figure 4.**
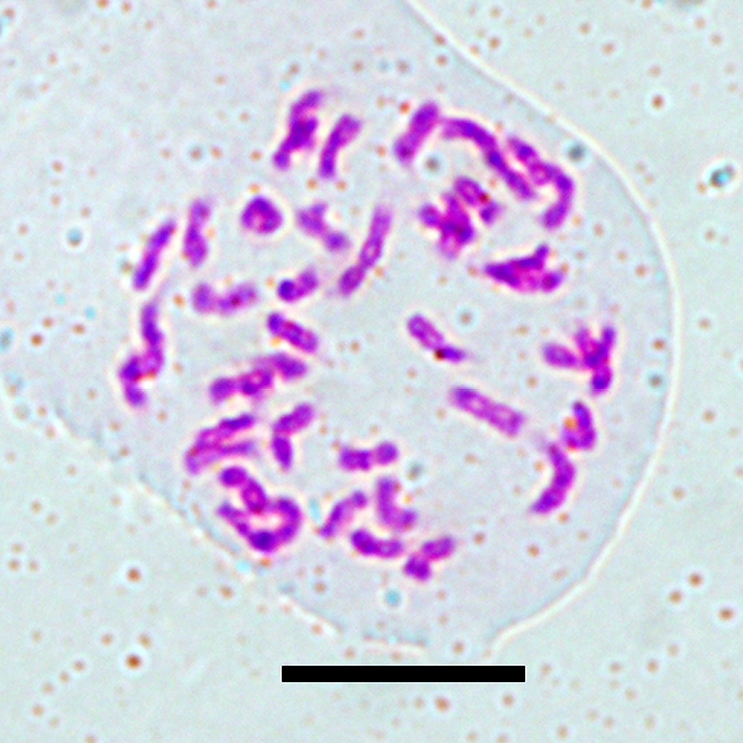
The chromosomes at metaphase in a root tip cell of *Polygala fallax*, 2n = 40. Scale bar: 10 µm.

For cytological study, young root tips (1–2 cm long) from five individual plants were collected and 20 metaphase plates were counted totally. All examined metaphase plates consistently showed 40 chromosomes. So *P. fallax* has the somatic chromosome number of 2n = 2x = 40 (Fig. 4) and the primary basic chromosome number is n = x = 20.

## Discussion

A genome encompasses all the information required for an individual’s development and functioning. Chromosome number, ploidy, and genome size estimates in angiosperms have been widely used to better understand their genome information (Choi et al. 2019; Kim et al. 2024). Chromosome number of *Polygala fallax*, 2n = 2x = 40, was reported for the first time in this study. Previous chromosomal data of Polygalaceae reveals a great diversity in the chromosome numbers and the genus *Polygala* does not have a fixed primary basic chromosome number (Bolkhovskikh et al. 1969; Moore 1973; Table 4). After checking the reported chromosome number data in the CCDB (Rice and Mayrose 2023), only one species, *P. curtissii* A. Gray, 1867, has the same chromosome number as *P. fallax*. This indicates that the chromosome number of *P. fallax* is special and different from most of the species within the genus (Lewis and Davis 1962; Bolkhovskikh et al. 1969; Moore 1973; Rice and Mayrose 2023).

**Table 4.** Comparison of genome size and chromosome number of 13 species in *Polygala*.

**No**.	**Species**	**2C-DNA value (Gb/pg)**	**Chromosome number, 2n**
1	* Polygala tenuifolia*	0.80/0.82	38
2	* Polygala amara* subsp. *brachyptera*	0.82/0.84	28, 34
3	* Polygala angustifolia*	0.83/0.85	–
4	* Polygala calcarea* cv. *Lillet*	0.84/0.86	34
5	* Polygala vulgaris*	0.87/0.89	34
6	* Polygala sanguinea*	0.88/0.90	–
7	* Polygala microphylla*	1.03/1.05	32
8	* Polygala major*	1.05/1.07	34
9	* Polygala polygama*	1.28/1.31	28
10	* Polygala supina*	1.75/1.79	–
11	* Polygala fallax*	2.25-2.49/2.30-2.61	40
12	* Polygala vayredae*	2.65/2.71	28
13	* Polygala paucifolia*	3.03/3.10	34

–: No data were reported.

Genome size plays a crucial role in genome sequencing projects, as it determines the sequencing depth budget and offers an initial estimate for assessing the completeness of genome assembly (Mgwatyu et al. 2020). The genome size data of *Polygala* in the “Plant DNA C-values Database” (Leitch et al. 2019) and the chromosome numbers in the CCDB (Rice and Mayrose 2023) are shown in Table 4, together with the information of *P. fallax* and *P. tenuifolia* (Shi and Niu 2017). The comparative framework shows that *P. tenuifolia* has the smallest genome size and genome size of *P. fallax* is the third largest in the genus. The genome size of *P. fallax* is about three times larger than that of *P. tenuifolia* (2199.64–2490 Mb vs. 798.58 Mb), but it has only two more chromosomes than *P. tenuifolia* (Shi and Niu 2017; Meng et al. 2023). Data of other species also show that there is no significant correlation between chromosome number and genome size. For example, *P. paucifolia* has the largest genome size in the genus, while it possesses the same chromosome number (2n = 34) with *P. amara* subsp. *brachyptera*, which has a second smaller genome size (Temsch et al. 2010; Bai et al. 2012).

There are similar cases in other families, for instance, *Silene latifolia* Poiret, 1789 and *S. vulgaris* (Moench, 1794) Garcke, 1869 (Caryophyllaceae) are both diploid species and possess the same chromosome number (2n = 24), but differ in their genome size and mode of reproduction (Cegan et al. 2010). The lack of a direct correlation between chromosome number and genome size indicates that genome size is not strictly determined by chromosome count, and may also rely on the content of repetitive sequences and transposable element (Gregory 2005; Bennetzen and Wang 2014).

Genome size evolution in plants is a complex process shaped by various molecular mechanisms and evolutionary forces, leading to considerable variation across species. Formation of a large genome is primarily driven by whole-genome duplication events and the accumulation of repeated sequences, such as tandem repeats and transposable elements (Bennett and Leitch 2005; Niu et al. 2022; Duan et al. 2025). For instance, transposable elements, which can replicate and insert themselves into other genomic locations, can significantly contribute to genome expansion without changing the number of chromosomes (Lee and Kim 2014).

Besides the above reasons, the large genome size may also originate from ancient polyploidy followed by diploidization, which is strongly supported by previous genomic research (e.g. Vision et al. 2000; Conant and Wolfe 2008; Soltis and Soltis 2016). However, this phenomenon has not been reported in Polygalaceae. Until recently, there was no high-quality, chromosome-level reference genome for any Polygalaceae species. Except for the only one, that of *P. tenuifolia*, was published in 2023. This lack of data become a major obstacle for comparative genomics, gene family analysis, and evolutionary studies.

Genomic information has significant value on studying genetic resources of plant species, and it is especially crucial for elucidating synthesis mechanisms of various secondary metabolites. Meng et al. (2023) demonstrated that whole-genome duplications and tandem duplications play critical roles in the expansion of P450 and UGT gene families, greatly contributing to the synthesis of triterpenoid saponins, which were also considered as the main medicinal component of *P. fallax*. The complexities of how the large genome of *P. fallax* was formed and its precise biological function remain to be determined through future analyses. In this study, we present the genomic and cytological data of *P. fallax*, in order to provide an important reference for whole-genome sequencing and the selection of assembly strategies in subsequent steps.

## Supplementary materials

Supplementary material 1 Additional figure https://doi.org/10.3897/compcytogen.20.184176.suppl1 Data type docx https://binary.pensoft.net/file/1596550This dataset is made available under the Open Database License (http://opendatacommons.org/licenses/odbl/1.0/). The Open Database License (ODbL) is a license agreement intended to allow users to freely share, modify, and use this Dataset while maintaining this same freedom for others, provided that the original source and author(s) are credited. Lin Fu, Ziyue Liu, Lina Han, Ting Zhang, Yan Jiang, Denis A. Krivenko, Jin Hu, Hongfeng Chen, Lei Duan
