# Supplementary figures and images for: Genome survey and chromosome number determination of Polygala fallax (Polygalaceae), an endemic medicinal plant from southern China
Source: Comp Cytogenet. 2026 Apr 15;20:127–39. doi: 10.3897/compcytogen.20.184176 (PMC13103666; doi:10.3897/compcytogen.20.184176)

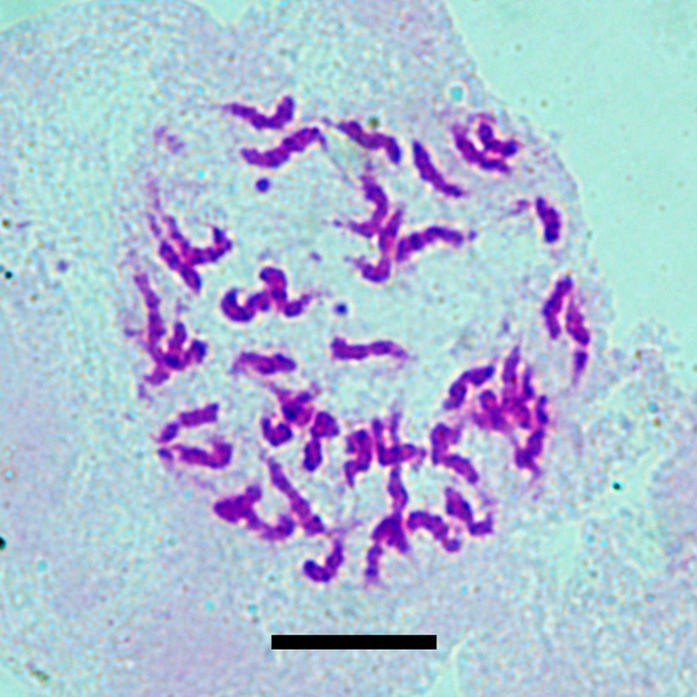

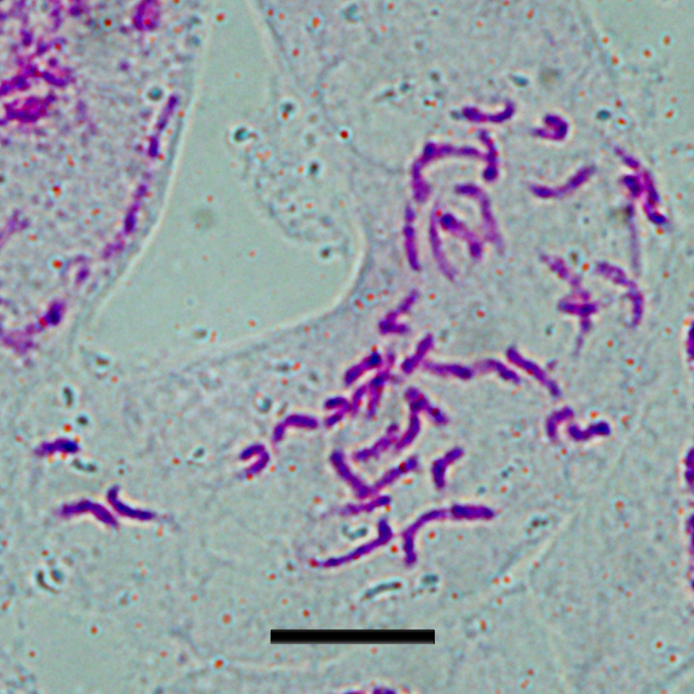

Supplement: Supplementary material 1 — Additional figure [file comparative_cytogenetics-20-127_article-184176__-s001.docx]
